# Memory and synaptic plasticity are impaired by dysregulated hippocampal O-GlcNAcylation

**DOI:** 10.1038/srep44921

**Published:** 2017-04-03

**Authors:** Yong Ryoul Yang, Seungju Song, Hongik Hwang, Jung Hoon Jung, Su-Jeong Kim, Sora Yoon, Jin-Hoe Hur, Jae-Il Park, Cheol Lee, Dougu Nam, Young-Kyo Seo, Joung-Hun Kim, Hyewhon Rhim, Pann-Ghill Suh

**Affiliations:** 1 School of Life Sciences, Ulsan National Institute of Science and Technology, Ulsan 44919, Republic of Korea; 2 Aging Research Center, Korea Research Institute of Bioscience and Biotechnology (KRIBB), Daejeon 34141, Republic of Korea; 3 Center for Neuroscience, Brain Science Institute, Korea Institute of Science and Technology (KIST), Seoul 136-791, South Korea; 4 Department of Neuroscience, University of Science and Technology (UST), Daejeon 305-333, South Korea; 5 Department of Life Science, Pohang University of Science and Technology, Pohang, Gyungbuk 790-784, Republic of Korea; 6 Korea Basic Science Institute, Gwangju 61186, Republic of Korea

## Abstract

O-GlcNAcylated proteins are abundant in the brain and are associated with neuronal functions and neurodegenerative diseases. Although several studies have reported the effects of aberrant regulation of O-GlcNAcylation on brain function, the roles of O-GlcNAcylation in synaptic function remain unclear. To understand the effect of aberrant O-GlcNAcylation on the brain, we used *Oga*^+/−^ mice which have an increased level of O-GlcNAcylation, and found that *Oga*^+/−^ mice exhibited impaired spatial learning and memory. Consistent with this result, *Oga*^+/−^ mice showed a defect in hippocampal synaptic plasticity. *Oga* heterozygosity causes impairment of both long-term potentiation and long-term depression due to dysregulation of AMPA receptor phosphorylation. These results demonstrate a role for hyper-O-GlcNAcylation in learning and memory.

O-GlcNAcylation is a posttranslational modification involving attachment of beta-*N*-acetylglucosamine (GlcNAc) to Ser/Thr residues. The addition and removal of O-GlcNAc to target proteins is regulated by O-GlcNAc transferase (OGT) and O-GlcNAcase (OGA), respectively^1^, and both OGT and OGA are abundantly expressed in the brain^2^,^3^. The roles of O-GlcNAcylation have been extensively investigated in aging-associated neurodegenerative diseases, such as Alzheimer’s disease and Parkinson’s disease^4^,^5^. Several aggregation-prone proteins involved in neurodegeneration are O-GlcNAcylated, including Tau^6^,^7^ and α-synuclein^8^,^9^. Elevated O-GlcNAcylation prevents protein aggregation and slows neurodegeneration^4^,^5^. β-amyloid precursor protein (APP) involved in amyloid plaque formation is also O-GlcNAcylated^10^. Increased total O-GlcNAcylation attenuates the production of oligomeric Aβ, the main component of senile plaques, by lowering the activity of γ-secretase^11^. Oligomeric forms of amyloid-β (Aβ) are known to acutely increase excitatory synaptic transmission and interfere with synaptic plasticity^12^,^13^. Previous evidence suggests that aberrant regulation of O-GlcNAcylation may contribute to the impaired synaptic plasticity in neurodegeneration.

O-GlcNAcylation is particularly enriched in neuronal synapses^14^,^15^, and proteomic studies have identified many postsynaptic density proteins modified by O-GlcNAc^8^,^16^. In addition, the activity of the brain OGT is ten-fold higher than that of peripheral tissues^2^. Various neuronal proteins are O-GlcNAcylated and involved in synaptic functions^1^,^17^. GluA2, a subunit of glutamatergic α-amino-3-hydroxy-5-methyl-4-isoxazole propionate (AMPA) receptors, interacts with OGT, and is O-GlcNAcylated. Although phosphorylation of GluA2 is not affected by increased O-GlcNAcylation, O-GlcNAcylation of GluA2 is required for synaptic plasticity^18^. Furthermore, synapsin I, a synaptic vesicle-associated protein, is O-GlcNAcylated, with suggested roles in the localization and function of synapsin I^19^,^20^. O-GlcNAcylation of synapsin I has been implicated in the modulation of synaptic plasticity. In addition, O-GlcNAcylation is present on proteins important for neuronal signaling. Calcium/calmodulin-dependent kinase II (CaMKII), CaMKIV, and the transcription factor cyclic adenosine monophosphate (AMP)–response element binding protein (CREB) are O-GlcNAcylated, which influences synaptic plasticity in the hippocampus^21^,^22^,^23^. Although it is clear that O-GlcNAcylation is abundant in synapses and that O-GlcNAcylation affects synaptic plasticity and learning and memory in the hippocampus, past studies have used different methods to modulate O-GlcNAcylation levels, resulting in conflicting results. Decreased O-GlcNAc levels by alloxan treatment (OGT inhibitor) impairs high-frequency stimulation (HFS)-induced long-term potentiation (LTP) in the Schaffer Collateral (SC)-CA1 Pathway^24^. In contrast, the elevation of O-GlcNAcylation induced by Thiamet-G (OGA inhibitor) inhibits HFS-LTP and impairs hippocampal learning^18^.

Here we assessed how chronic elevations of O-GlcNAcylation in the hippocampus affect synaptic function, behavioral traits, and spatial learning and memory, using *Oga*^+/−^ mice with constitutively increased O-GlcNAc levels.

## Results

### *Oga* ^+/−^ brains have normal morphology and dendritic spine density

**Figure 1:**
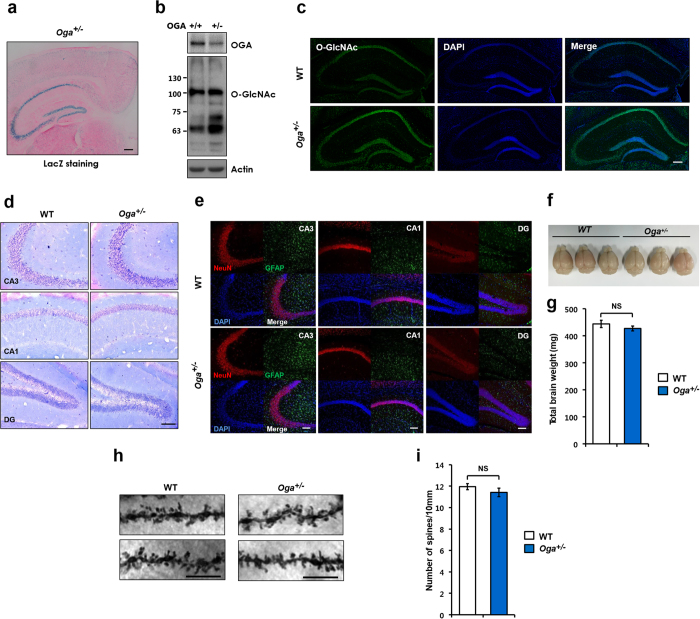
Normal morphological features and dendritic spine density in *Oga*^+/−^ brains (**a**) Beta galactosidase (LacZ) staining of the *Oga*^+/−^ adult brain, confirming the expression pattern of OGA in the hippocampus. Scale bar, 200 μm. (**b**) Immunoblot analysis showing elevated O-GlcNAcylation in *Oga*^+/−^ hippocampal lysates compared with that in the WT hippocampal lysates. (**c**) Representative images of hippocampal neurons from WT and *Oga*^+/−^ mice immunolabeled for O-GlcNAc (green). Scale bar, 200 μm. (**d**) Nissl staining of the hippocampus from coronal brain sections. Scale bar, 100 μm. (**e**) Immunostaining for a neuronal cell marker (neuronal nuclei; NeuN), glial marker (glial fibrillary acidic protein; GFAP), and nuclei (4′,6-diamidino-2-phenylindole; DAPI) in the hippocampus of WT and *Oga*^+/−^ mice at eight weeks of age. Three sections were obtained from three mice. Scale bar, 100 μm. (**f**) Representative pictures of brain tissues. (**g**) Whole-brain weight (without skull) isolated from the WT and *Oga*^+/−^ mice at eight weeks of age (n = 5). (**h**) Representative images of dendritic segments of Golgi-stained CA1 pyramidal neurons from WT and *Oga*^+/−^ mice (scale bar = 10 μm) (**i**) Spine number was quantified along a 10 μm segment from the primary apical dendritic branch origin of Golgi-impregnated CA1 pyramidal neurons (n = 15–20 dendrites from three mice). Error bars represent ± standard error of the mean (SEM). NS: not significant (unpaired *t*-test). Full-length blots/gels are presented in Supplementary Figure S4.

Consistent with previous studies showing enriched expression of O-GlcNAc cycling enzymes, OGT and OGA, we detected high levels of O-GlcNAcase expression in the hippocampus, which was visualized by beta galactosidase (LacZ) staining of an *Oga*^+/−^ brain section (Fig. 1a). To assess the effect of increased O-GlcNAcylation on hippocampus-dependent function, we used *Oga*^+/−^ mice with chronically elevated O-GlcNAcylation. The hippocampal lysates prepared from *Oga*^+/−^ mice showed an increase in the overall O-GlcNAcylation levels (Fig. 1b). To verify the elevation of O-GlcNAcylation in the hippocampus, we used immunohistochemistry with an anti-O-GlcNAc antibody. As expected, increased immunoreactivity was observed throughout all regions of the hippocampus in *Oga*^+/−^ mice compared to WT. (Fig. 1c). Next, we tested whether *Oga* heterozygosity leads to morphological changes in the brain. Morphological analysis of neurons in the hippocampus by Nissl staining revealed that the *Oga*^+/−^ hippocampus shows no morphological changes in hippocampal CA1, CA3, or dentate gyrus (DG) (Fig. 1d). In addition, we found that there were no differences in the numbers of cells immunostained for the neuronal marker neuronal nuclei (NeuN) and for the astrocyte marker glial fibrillary acidic protein (GFAP) in the hippocampal CA1, CA3, or DG (Fig. 1e). The average brain weight also was not affected by *Oga* heterozygosity (Fig. 1f and g). Lastly, dendritic spine density was not altered in the *Oga*^+/−^ hippocampal CA1 pyramidal neurons (Fig. 1h and i). These results together suggest that synaptic development and hippocampal structure are not affected by the elevation of O-GlcNAcylation.

### *Oga* ^+/−^ mice display impaired spatial learning and memory

**Figure 2:**
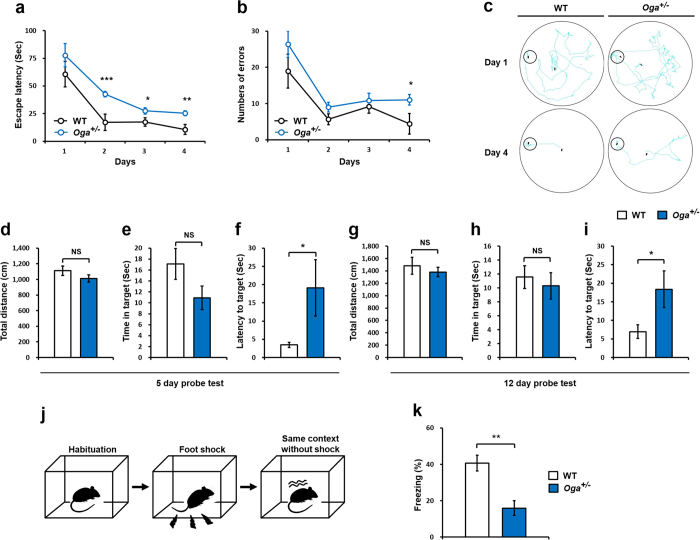
Spatial learning memory deficits in *Oga*^+/−^ mice (**a**) Latency in attaining the goal and (**b**) number of errors committed before reaching the goal in the Barnes maze task. (WT, n = 9; *Oga*^+/−^, n = 9, one-way ANOVA followed by the Tukey’s test to unpaired t-test) (**c**) Representative traces of WT and *Oga*^+/−^ mice in the Barnes maze task. (**d**) Total distance, (**e**) time spent in the target area, and (**f**) time of latency to attaining the target hole during a probe trial on day 5. (**g**) Total distance, (**h**) time spent in the target area, and (**i**) time of latency to attain the target hole during a probe trial on day 12. (WT, n = 9; *Oga*^+/−^, n = 9) (**j**) Schematic diagram of the context-dependent fear conditioning procedure (**k**) Percentage time freezing during the 3 min of the context test 24 h after fear conditioning. (WT, n = 9; *Oga*^+/−^, n = 8). Error bars represent ± standard error of the mean (SEM). NS: not significant, ***P < 0.001, **P < 0.01, *P < 0.05 (unpaired *t*-test).

Various neuronal proteins involved in synaptic function and learning and memory are known to be O-GlcNAcylated^8^,^9^,^15^. To assess whether hyper-O-GlcNAcylation affects hippocampal-dependent spatial learning and memory, we employed the Barnes circular maze test. In this test, mice were trained to escape a brightly lighted circular field by discovering the escape hole at its periphery. Compared with wild-type (WT) mice, *Oga*^+/−^mice showed impaired learning performance during four days of training (Fig. 2a–c). To assess memory formation, we performed probe trials on days 5 and 12. WT and *Oga*^+/−^mice performed similarly during the probe trials when total distance was measured (Fig. 2d and g). *Oga*^+/−^ mice exhibited increased latency to the target region during probe trials (Fig. 2f and i). However, no significant difference were observed in the time spent in the target region between WT and *Oga*^+/−^ mice (Fig. 2e and h). To further verify the impairment in spatial learning and memory of *Oga*^+/−^ mice, we performed a context fear conditioning test. Compared with WT mice, *Oga*^+/−^ mice failed to retain fear memory 24 h after fear conditioning (Fig. 2j and k). Our data indicate that proper removal of O-GlcNAc modification by OGA is required for hippocampal-dependent spatial learning and memory.

To examine motor coordination in these mice, we tested motor performance using the rotarod task. *Oga*^+/−^ mice did not display a defect in motor function during the rotarod test (Fig. S1A). During the open field test, *Oga*^+/−^ mice showed normal locomotor activities (Fig. S1B and C). Anxiety-related behaviors were also tested using the elevated plus maze. Compared with WT mice, *Oga*^+/−^ mice exhibited no significant differences in the number of entries to the open arms and amount of time spent in the open arms (Fig. S1D–G). Considered collectively, these data indicate that *Oga*^+/−^ mice show normal locomotor activity and anxiety levels.

### Glutamatergic and GABAergic synaptic transmission in the hippocampus is normal in *Oga* ^+/−^ mice

**Figure 3:**
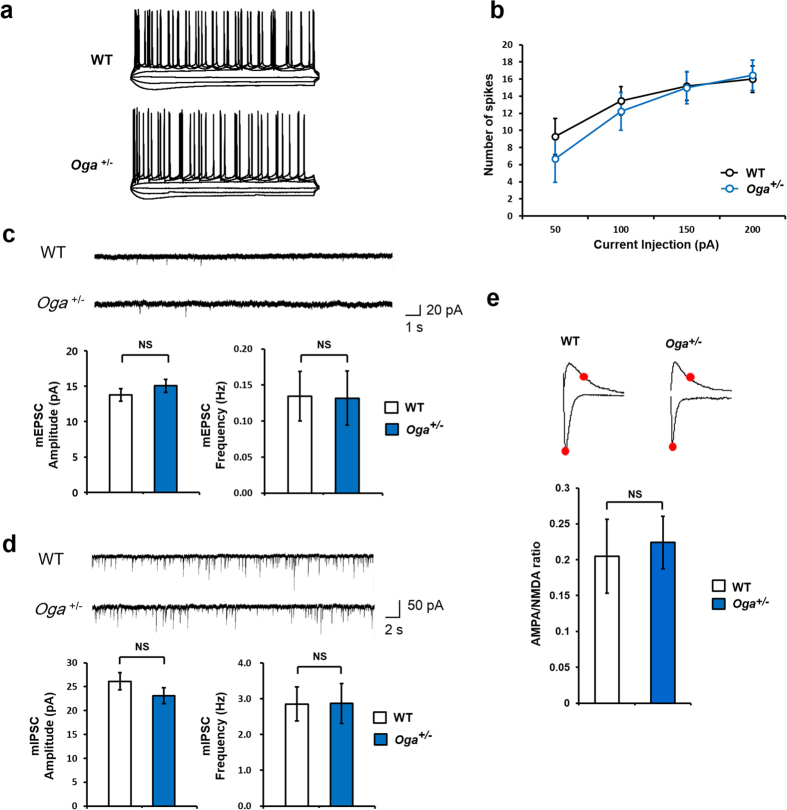
Normal synaptic transmission in *Oga*^+/−^ mice. (**a**) Representative traces of action potentials triggered by −100–200 pA current injection in the hippocampal CA1 region of WT and *Oga*^+/−^ mice. (**b**) Number of action potentials trigged by the injection of current at different levels (WT, n = 11; *Oga*^+/−^, n = 9; unpaired *t*-test, not significant) (**c**) Representative mEPSC traces from WT and *Oga*^+/−^ hippocampal CA1 pyramidal neurons (upper). Average values for mEPSC amplitude (lower left) and frequency (lower right) (WT, n = 9; *Oga*^+/−^, n = 10; unpaired *t*-test, NS: not significant) (**d**) Representative mIPSC traces from WT and *Oga*^+/−^ hippocampal CA1 pyramidal neurons (upper). Average values for mIPSC amplitude (lower left) and frequency (lower right). (WT, n = 10; *Oga*^+/−^, n = 8; unpaired *t*-test, NS: not significant) (**e**) AMPA/NMDA current ratio (WT, n = 20; *Oga*^+/−^, n = 17; Mann-Whitney U test, NS: not significant).

We next explored the effect of heterozygous loss of *Oga* on intrinsic neuronal excitability and excitatory synaptic transmission in hippocampal CA1 pyramidal neurons. Excitability was tested by injecting step depolarizing currents, and we found that intrinsic excitability of hippocampal CA1 pyramidal neurons remains unchanged in *Oga*^+/−^ mice (Fig. 3a and b). The frequency and amplitude of miniature excitatory and inhibitory postsynaptic currents (mEPSCs and mIPSCs, respectively) in hippocampal CA1 pyramidal neurons were also comparable in WT and *Oga*^+/−^ pyramidal neurons (Fig. 3c and d), indicating that basal synaptic responses are not affected in *Oga*^+/−^ mice. In addition, we measured the ratio of AMPA to N-methyl-D-aspartate (NMDA) receptor-mediated synaptic currents in SC−CA1 synapses, and the AMPA/NMDA ratio was similar between WT and *Oga*^+/−^ synapses (Fig. 3e). These results suggest that *Oga* heterozygosity does not affect basal SC−CA1 synaptic transmission or short-term plasticity.

### Impaired NMDA receptor (NMDAR)-dependent synaptic plasticity in *Oga* ^+/−^ mice

**Figure 4:**
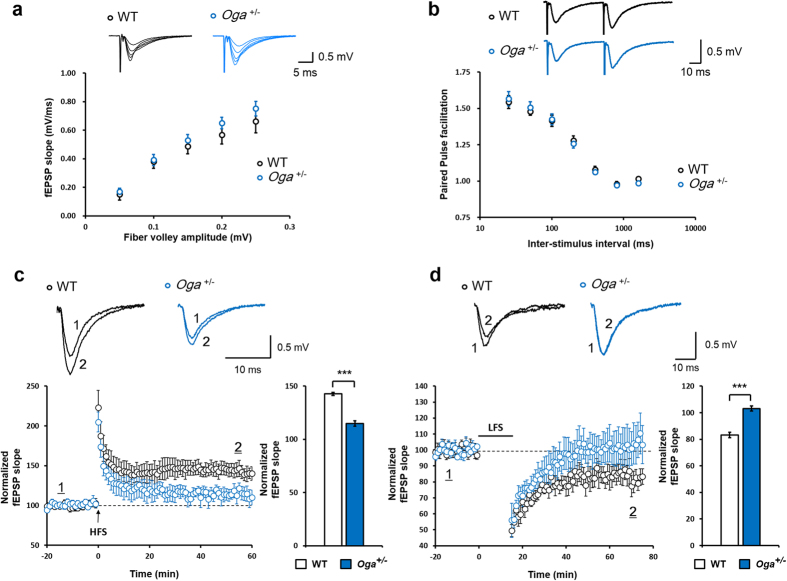
Impaired long-term potentiation (LTP) and long-term depression (LTD) in *Oga*^+/−^ mice. (**a**) Input-output curves for basal synaptic transmission in area CA1 of the hippocampus. Representative traces are shown for the input (fiber volley) and the output (field excitatory postsynaptic potential; fEPSP). (WT, n = 9; *Oga*^+/−^, n = 10; unpaired *t*-test, not significant) (**b**) Paired-pulse facilitation (PPF) in WT and *Oga*^+/−^ hippocampal CA1 pyramidal neurons. Representative traces from WT and *Oga*^+/−^ at 50 ms interstimulus interval are shown. (WT, n = 8; *Oga*^+/−^, n = 10; unpaired *t*-test, not significant) (**c**) High frequency stimulation (HFS)-induced LTP (WT, n = 10; *Oga*^+/−^, n = 9). Traces show averaged fEPSP indicated with 1 and **2**. A bar graph is depicted 50 min after LTP. (**d**) LFS-induced LTD (WT, n = 7; *Oga*^+/−^, n = 8). Traces show averaged fEPSP indicated with 1 and **2**. A bar graph is depicted 50 min after LTD. Error bars represent ± standard error of the mean (SEM). ***P < 0.001 (unpaired *t*-test).

Previous studies investigating the effects of increased O-GlcNAcylation on synaptic plasticity have generated conflicting results^18^,^24^. Therefore, we next assessed whether increased O-GlcNAcylation resulting from *Oga* haploinsufficiency alters synaptic plasticity. The slope of the field excitatory postsynaptic potential (fEPSP) to fiber volley amplitudes (input-output curves) was not changed in *Oga*^+/−^ mice (Fig. 4a). Presynaptic release probability, as measured by paired-pulse facilitation (PPF), also remained unaffected in *Oga*^+/−^ mice (Fig. 4b). When we measured NMDAR-mediated LTP in *Oga*^+/−^ mice, the magnitude of LTP induced by high-frequency stimulation (HFS) at the SC−CA1 pathway was reduced compared to WT mice (Fig. 4c). Moreover, in the same hippocampal pathway, low-frequency stimulation (LFS)-induced long-term depression (LTD) was impaired in *Oga*^+/−^ mice in comparison to WT mice (Fig. 4d). These results suggest that the removal of O-GlcNAcylation mediated by OGA is required for NMDAR-dependent LTP and LTD at SC−CA1 synapses.

### Impaired modulation of AMPA receptor during LTP/LTD in *Oga* ^+/−^ mice

**Figure 5:**
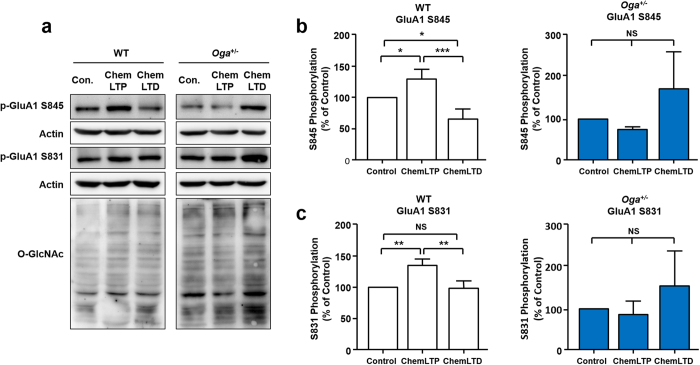
Deficit in chemically induced LTP and LTD in *Oga*^+/−^ mice. (**a**) Representative immunoblots showing effects of chemical LTP and LTD induction on AMPAR subunits GluA1 S845, S831 phosphorylation. Acute hippocampal slices were stimulated with either glycine (200 μM) for chemical LTP or NMDA (20 μM) for chemical LTD. Levels of phospho-S845 and phospho-S831 of GluA1, and levels of total O-GlcNAc-modified proteins were analyzed by immunoblotting. (**b**) Chemical LTP and LTD significantly increases and decreases the levels of GluA1 S845 phosphorylation, respectively in WT (n = 4, normalized to control). However, acute hippocampal slices from *Oga*^+/−^ mice failed to exhibit a significant change in GluA1 S845 phosphorylation following chemical LTP and LTD induction (n = 3, normalized to control). One-way ANOVA followed by the Tukey’s test was used. (**c**) Chemical LTP significantly increases the levels of GluA1 S831 phosphorylation in WT (n = 3, normalized to control), but not in *Oga*^+/−^ mice (n = 3, normalized to control). Error bars represent ± standard error of the mean (SEM). NS: not significant, *p < 0.05, **p < 0.01, ***p < 0.001; one-way ANOVA followed by the Tukey’s test. Full-length blots/gels are presented in Supplementary Figure S4.

Regulation of AMPA receptor trafficking is crucial for controlling the strength of synaptic transmission during LTP/LTD. In particular, phosphorylation of GluA1 AMPAR subunit at S845 and S831 play key roles in AMPA receptor trafficking and synaptic plasticity^25^. We thus decided to examine whether GluA1 phosphorylation is altered in response to chemically induced LTP and LTD in *Oga*^+/−^ hippocampus. We briefly stimulated hippocampal slices from WT and *Oga*^+/−^ mice with glycine for LTP or NMDA for LTD^26^,^27^. WT hippocampal slices showed elevated phosphorylation of the S845 and S831 GluA1 in chemical LTP, and the phosphorylation of the S845 GluA1 was decreased in chemical LTD. However, in *Oga*^+/−^ hippocampal slices, phosphorylation of the S845 and S831 GluA1 were not properly regulated following chemical LTP or LTD (Fig. 5a–c). These results indicate that *Oga* heterozygosity impairs the proper regulation of AMPA receptor phosphorylation during synaptic plasticity.

Diverse neuronal proteins and signaling mediators are O-GlcNAcylated and involved in synaptic functions. For example, AMPA receptor subunit GluA2 is O-GlcNAcylated by OGT^18^, and CaMKII, CaMKIV, and CREB whose alternation of activity affects synaptic plasticity^28^,^29^, are also O-GlcNAcylated^21^,^22^,^23^. *Oga* heterozygosity did not alter the basal phosphorylation or total protein levels AMPA receptor subunits (GluA1, GluA2) and NMDA receptor subunits (GluN1, Glun2A, GluN2B) (Fig. S2A). In addition, no difference was observed in phosphorylation of CaMKII, CaMKIV, or CREB between the hippocampus of WT and *Oga*^+/−^ mice (Fig. S2B).

## Discussion

Although increasing evidence has been generated by various studies regarding the significance of O-GlcNAcylation in regulating synaptic functions, the different experimental designs used have resulted in conflicting conclusions on the impact of changing the levels of O-GlcNAcylation. Here we used mice with a heterozygous loss-of-function mutation in OGA which have elevated O-GlcNAc levels. We found that OGA is highly expressed in the hippocampus, suggesting that O-GlcNAc modification of neuronal proteins is closely related to hippocampus-dependent functions. *Oga*^+/−^ mice exhibited impaired synaptic plasticity in the hippocampus at SC-CA1 synapses, dysregulated phosphorylation of AMPA receptor subunit GluA1 in chemically induced LTP and LTD, and deficits in hippocampus-dependent learning and memory. This result together demonstrates that increased levels of O-GlcNAcylation lead to altered synaptic plasticity in the hippocampus, which may underlie the impairment of learning and memory observed in *Oga*^+/−^ mice.

Several studies have shown that synaptic plasticity is variably affected by O-GlcNAcylation. Tallent *et al*. showed that the elevation of O-GlcNAcylation induced by OGA inhibitor (9d) decreases PPF and increases LTP induction, and suggested that the elevation of O-GlcNAcylation facilitated LTP by modulating the interplay between phosphorylation and O-GlcNAcylation of signaling molecules, such as synapsin I/II, ERK, and CaMKII^24^. In the same study, the authors also reported that reduced O-GlcNAcylation with OGT inhibitor (Alloxan) prevents LTP induction^24^. However, contrary to this result, Kanno *et al*. found that Alloxan enhances hippocampal SC-CA1 LTP by regulating AMPA receptor trafficking^30^. Taylor *et al*. also showed that acutely elevated O-GlcNAcylation by OGA inhibitor (Thiamet-G) or glucosamine induces LTD, but impairs LTP at CA3-CA1 synapses, which also led to a deficit in novel object recognition^18^. Each study mentioned above used different methods to change the levels of O-GlcNAcylation. Alloxan is known as a weak OGT inhibitor and thus likely to have off-target effects^31^,^32^. Furthermore, as we previously reported, the OGA inhibitor (Thiamet-G) increases the levels of OGA expression^33^,^34^. Glucosamine also affects various intracellular signaling pathways^35^,^36^,^37^. Each experiment was performed in acutely elevated O-GlcNAcylation by pretreatment of OGA inhibitors or glucosamine. The different experimental designs might have resulted in conflicting results. Previously, the discrepancy in the effect of dysregulated O-GlcNAcylation was also observed in other intracellular signaling pathways and physiological functions^33^,^34^,^38^. Despite this discrepancy, earlier studies suggest that dysregulated O-GlcNAcylation can affect synaptic plasticity. Here, we used OGA heterozygous mice that have elevated O-GlcNAcylation levels. *Oga*^+/−^ hippocampus displayed impaired regulation of AMPAR GluA1 phosphorylation which plays an important role in mediating AMPAR trafficking during synaptic plasticity. Although Taylor *et al*. showed that GluA1 is not O-GlcNAcylated^18^, the phosphorylation of GluA1 can be indirectly regulated by activation of upstream signaling molecules, including protein kinase C (PKC), CaMKII, and protein kinase A (PKA)^39^,^40^,^41^. Importantly, both PKC and CaMKII are modified by O-GlcNAcylation^21^,^42^, and the dynamic interplay between O-GlcNAcylation and phosphorylation in neurons was shown to be involved in hippocampal synaptic plasyticity^24^. Activation of PKC or PKA reduces global O-GlcNAc levels in cytoskeletal fraction of cultured cerebellar neurons^43^. We speculate that GluA1 phosphorylation might be affected by altered O-GlcNAcylation levels. However, we cannot rule out the possibility that *Oga* heterozygosity affects multiple signaling pathways involved in hippocampal LTP and LTD.

ROS play an important role in synaptic plasticity^44^ by regulating synaptic plasticity-related signaling molecules, receptors, and channels^45^,^46^,^47^. Importantly, O-GlcNAcylation have been shown to affect ROS generation^48^. In addition, forkhed box O1 (FoxO1), a regulator of the transcription of the oxidative stress responsive enzymes catalase and MnSOD (SOD2), is O-GlcNAcylated^49^. We therefore examined whether ROS levels are affected in *Oga*^+/−^ hippocampus compared to WT hippocampus. Despite *Oga* heterozygosity, the ROS levels were not altered in *Oga*^+/−^ hippocampus (Fig. S3).

Aging is associated with impairments in cognitive and synaptic function^50^. Dysfunction of the aging brain is not caused by neuronal loss^51^ but by specific alterations in neuronal morphology, cell-cell interactions, and gene expression^50^. The hippocampus appears to be particularly vulnerable to the effects of aging on cognitive function and synaptic plasticity. O-GlcNAcylation and its regulatory enzymes are highly detected in the hippocampus^52^. O-GlcNAcylation modulates neuronal cell signaling processes and gene expression, which is critical for proper neuronal function^1^,^53^. Interestingly, we previously reported that the brains of older mice show significantly increased levels of O-GlcNAcylation compared with those in younger mice^3^. However, the mechanism underlying the chronic elevations in O-GlcNAcylation on brain aging remains unknown. Based on our observations, in the normally aged brain, we speculate that chronically elevated O-GlcNAcylation contributes to impairment of synaptic plasticity and learning and memory.

## Methods

### Mice

*Oga*^+/−^ mice (C57BL/6J) were generated as described previously^3^. All mice were housed under a 12-hour light/dark cycle and given *ad libitum* access to food and water. All experimental protocols were approved by Institutional Animal Care and Use Committee of the Ulsan National Institute of Science and Technology (UNISTIACUC-14–018) and all methods were performed in accordance with the relevant guidelines and regulations.

### Barnes maze

The paradigm of Barnes circular maze consists of white circular platform (92 cm diameter), with 20 evenly spaced holes (5 cm diameter) located 7.5 cm from the perimeter and is elevated 100 cm above the floor. Several spatial cues with distinct shapes were placed near the walls of the testing room. A black target box (20 × 10 × 10 cm) was placed under one hole. The mice were encouraged to find this box by aversive noise (85 dB) on the platform. Barnes maze were run for 4 consecutive days, and 3 trials were carried out each day with 20 min inter-trial intervals. The mouse was allowed to search for the target box for 3 min. Distance, latency, and numbers of errors to reach the target hole were recorded during training trials by video tracking software. On day 5, a probe test was performed without the escape box. Mice were allowed to freely find the target box for 3 min. Time spent around each hole, total distance travelled, and the latency to find target hole were recorded.

### Golgi staining

Brains from 8-week-old mice were processed with the FD Rapid GolgiStain™ Kit (NeuroTechnologies) according to the instructions of the manufacturer. Images of dendritic spines (apical dendrites of CA1 pyramidal neurons) were acquired using an Olympus Cell^TIRF Xcellence microscope in UNIST-Olympus Biomed Imaging Center (UOBC).

### Chemical LTP and LTD induction

Acute hippocampal slices (300-μm thick) from WT or *Oga*^+/−^ mice (8–10 weeks) were prepared in a sucrose-cutting buffer containing (in mM) 234 sucrose, 2.5 KCl, 1.25 NaH_2_PO_4_, 24 NaHCO_3_, 11 glucose, 10 MgSO_4_, 0.5 CaCl_2_ bubbled with 95% O_2_ and 5% CO_2_. The slices were recovered at 35 °C for one hour in a recovery buffer containing (in mM) 124 NaCl, 3 KCl, 1.25 NaH_2_PO_4_, 26 NaHCO_3_, 10 glucose, 6.5 MgSO_4_, 1 CaCl_2_ bubbled with 95% O_2_ and 5% CO_2_. Following the recovery, the slices were further incubated at 37 °C for one hour in an extracellular fluid containing (in mM) 125 NaCl, 2.5 KCl, 1 MgCl_2_, 2 CaCl_2_, 33 glucose, 25 HEPES, and then treated with 20 μM D-AP5 and 0.5 μM TTX for 20 min. The slices were subsequently treated with 3 μM strychnine, 20 μM bicuculline and 200 μM glycine for 10 min to induce chemical LTP, or with 20 μM NMDA for 3 min to induce chemical LTD in a Mg-free extracellular fluid, and transferred back to a regular extracellular fluid for 30 min prior to sample collection.

### Statistical analysis

The Student’s unpaired T-test or non-parametric Mann–Whitney U-test was used to compare two independent groups. For multiple comparisons, a one-way repeated measures ANOVA with Tukey’s *post hoc* test was utilized, as specified in the Figure legends. All data are expressed as the mean ± SEM and significance indicated by *P < 0.05, **P < 0.01, and ***P < 0.001.

## Additional Information

**How to cite this article:** Yang, Y. R. *et al*. Memory and synaptic plasticity are impaired by dysregulated hippocampal O-GlcNAcylation. *Sci. Rep.* **7**, 44921; doi: 10.1038/srep44921 (2017).

**Publisher's note:** Springer Nature remains neutral with regard to jurisdictional claims in published maps and institutional affiliations.

## Supplementary Material

Supplemental Information and Figures
