# Supplementary material for: Memory and synaptic plasticity are impaired by dysregulated hippocampal O-GlcNAcylation
Source: Sci Rep. 2017 Apr 3;7:44921. doi: 10.1038/srep44921 (PMC5377249; doi:10.1038/srep44921)

Supplemental Information

Memory and synaptic plasticity are impaired by dysregulated hippocampal O-GlcNAcylation

Yong Ryoul Yang, Seungju Song, Hongik Hwang, Jung Hoon Jung, Su-Jeong Kim, Sora Yoon, Jin-Hoe Hur, Jae-Il Park, Cheol Lee, Dougu Nam, Young-Kyo Seo, Joung-Hun Kim, Hyewhon Rhim, Pann-Ghill Suh

Supplemental experimental procedures

Rotarod test

Mice were placed in a neutral position on a cylinder of the rotarod apparatus (Letica Scientific Instruments, Barcelona). he speed of rotation is gradually increased from 4 to 35 rpm over the course of 5 min. Latency to fall was recorded for each mouse in three trials with 20-min intervals between trials.

Open field test

Open field testing was performed in the open field apparatus (60 × 60 cm), and the center zone line was 10 cm apart from the edge. After habituated in the test room, mice were released in the center-side square of the box in the beginning of assay, and mouse activities were measured with a video camera for 30 or 60 min, and analyzed by the SMART Video Tracking software (PanLab, Harvard Apparatus).

Elevated plus maze

Elevated plus maze test apparatus consisted of two open arms (30 × 5 cm) and two

closed arms of the same size by darkened plastic walls (30 cm × 5 cm × 15 cm), and a center area (5 × 5 cm), elevated to a height of 50 cm above the floor. Mice are allowed to freely explore the maze for 10 min. The number of arm entries and the amount of time spent in the open and closed arms, total distance traveled in each arm as well as total distance overall were analyzed by SMART software.

Contextual fear conditioning test

Contextual fear conditioning paradigm was followed by the previous study (Gao et al., 2010). Habituation was done for 2 days before behavioral training. For Contextual fear conditioning training, which consisted of a 3 min exposure of mice to the conditioning chamber (26 cm x 26 cm x 24 cm) followed by a foot shock (2 s, 0.8 mA). Mice were returned to their home cage 30 s after the shock delivery. On following day, mice were exposed the conditioning chamber during 3 min for retention test of contextual fear memory. Percent of time spent freezing during in the conditioning context was measured as the time duration of immobility with the exception of respiratory movements.

Primary antibodies

The following commercial antibodies were used for Western blotting or immunostaining: β-actin (MP Biomedicals, 691001), anti-O-GlcNAc (RL2)(Thermo, MA1-072), NeuroN (Millipore, MAB337), GFAP (Abcam, ab7260), p-CaMKII (Abcam, ab32678), CaMKII (Abcam, ab52476), p-Creb (Cell Signaling Technology, 9198), Creb (Cell Signaling Technology, 9197), p-GluA1 (Ser831) (Millipore, 04-823), p-GluA1 (Ser845) (Millipore, 04-1073), GluA1 (Millipore, 04-855), p-GluA2 (Ser880) (Millipore, 07-294), GluA2 (Millipore, MABN71), p-GluN2B (Ser1303) (Millipore, 04-1148), and GluN2B (NeuroMab, 75-097). Anti-OGT and OGA polyclonal antibodies had been previously generated and were used as

previously described(Song et al., 2008)

Immunohistochemistry

30 μm coronal brain sections from 8-week-old mice were air dried for 30min and rinsed in PBS 3times. After blocking with 5% normal goat serum (Santa Cruz), neuronal cells and glial cells were immunolabeled with anti-NeuN (1:500, Millipore) and anti-GFAP (1:500, abcam) antibodies respectively. O-GlcNAcylated proteins were reacted with the anti-O-GlcNAc antibody (RL2) (1:500, Thermo). After the washing, anti-mouse or anti- rabbit Alexa-568 conjugated IgG or anti-mouse Alexa-488 conjugated IgG (1:500, Invitrogen, Carlsbad, CA) antibodies were used.

Electrophysiology

Hippocampal slice (300μm thick) from WT and *Oga*$^{+/-}$ adult mice (8-9 weeks) were prepared and dissected using ice-cold buffer containing (in mM) 234 sucrose, 2.5 KCl, 1.25 NaH_2PO_4, 24 $NaHCO_3$, 10 glucose, 0.5 $CaCl_2$, and 10 $MgSO_4$, and bubbled with 95 % O2 and 5 % CO2. The slices were recovered 35°C for 1 hour and maintained at room temperature. Standard artificial CSF (in mM: 119 NaCl, 2.5 KCl, 1.25 NaH_2PO_4, 26 $NaHCO_3$, 10 glucose, 2.5 $CaCl_2$, 2 $MgSO_4$) was used in recording. Extracellular field EPSPs were recorded using glass pipette filled with ASCF. LTP was induced HFS protocol consisted of four strains of 100 Hz stimulation divided by 20 seconds. LTD was induced LFS protocol consisted of 1 Hz lasting 15 minutes. Mice used for LTD were 4-5 weeks old. To measure paired pulse facilitation (PPF), we apply interstimulus intervals of 25, 50, 100, 200, 400, 800 and 1600 ms.

To measure mEPSCs and mIPSCs, CA1 pyramidal cells were patched. Pipette was filled with internal solution composed (in mM) 115 K-gluconate, 10 KCl, 10 HEPES, 10 EGTA, 5 MgATP, 0.5 NaGTP for mEPSCs or 100 CsCl, 10 HEPES, 1 EGTA, 5 MgATP, 0.5 NaGTP

for mIPSCs. TTX (0.5 mM), bicuculline (20 mM), D-APV (20 mM) were added to ACSF for mEPSCs recording, and TTX (0.5 mM), DNQX (10 mM), D-APV (20 mM) were added to ACSF for mIPSCs recording. For measurement of neuronal excitability, pipette was filled with internal solution composed (in mM) 140 K-gluconate, 10 HEPES, 7 NaCl, 5 MgATP, 0.5 NaGTP.

Responses were recorded at holding potentials of -70 mV (for AMPAR-mediated responses) and +40 mV (for NMDAR-mediated responses) in ACSF with 100 uM PTX. AMPAR-mediated responses measured the peak amplitude of the EPSC. NMDAR-mediated responses were quantified the amplitude at 50 ms after stimulation. The pipette internal solution was comprised of (in mM) 130 Cesium methane sulfonate, 10 HEPES, 0.5 EGTA, 8 NaCl and 10 phosphocreatine, 2 Mg-ATP, 0.1 Na-GTP and 5 QX-314.

Analysis of reactive oxygen species (ROS)

ROS was measured using a commercial DCFDA-cellular ROS detection assay kit as manufacturer's instructions (Abcam, Cambridge, UK).

REFERENCES

Gao, J., Wang, W.Y., Mao, Y.W., Graff, J., Guan, J.S., Pan, L., Mak, G., Kim, D., Su, S.C., and Tsai, L.H. (2010). A novel pathway regulates memory and plasticity via SIRT1 and miR-134. Nature *466*, 1105-1109.

Song, M., Kim, H.S., Parka, J.M., Kim, S.H., Kim, I.H., Ryu, S.H., and Suh, P.G. (2008). O-GlcNAc transferase is activated by CaMKIV-dependent phosphorylation under potassium chloride-induced depolarization in NG-108-15 cells. Cell Signal *20*, 94-104.

Supplementary Figure S1

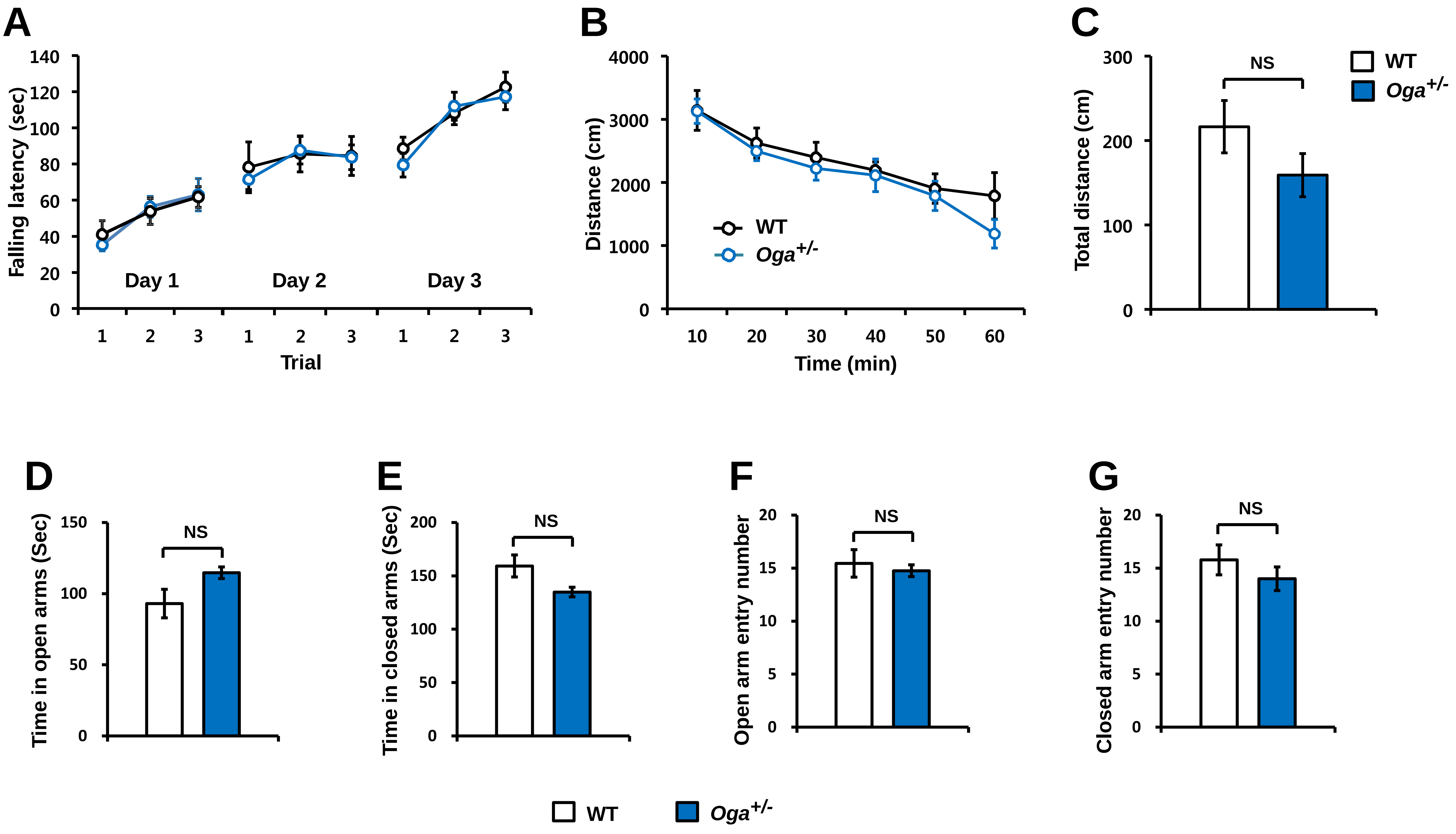

Figure S1. *Oga*$^{+/-}$ mice show normal locomotor activity and anxiety-like behavior (A) Rotarod test of wild-type (WT) and *Oga*$^{+/-}$ mice. (B, C) locomotor activity of WT and *Oga*$^{+/-}$ mice in the open field test (WT, n = 10; *Oga*$^{+/-}$, n = 10) (D–G) Elevated plus maze test of WT and *Oga*$^{+/-}$ mice. Time spent in (D) open arms and (E) closed arms. Entry number in (F) open arms and (G) closed arms. (WT, n = 10; *Oga*$^{+/-}$, n = 10; unpaired *t*-test, not significant)

Supplementary Figure S2

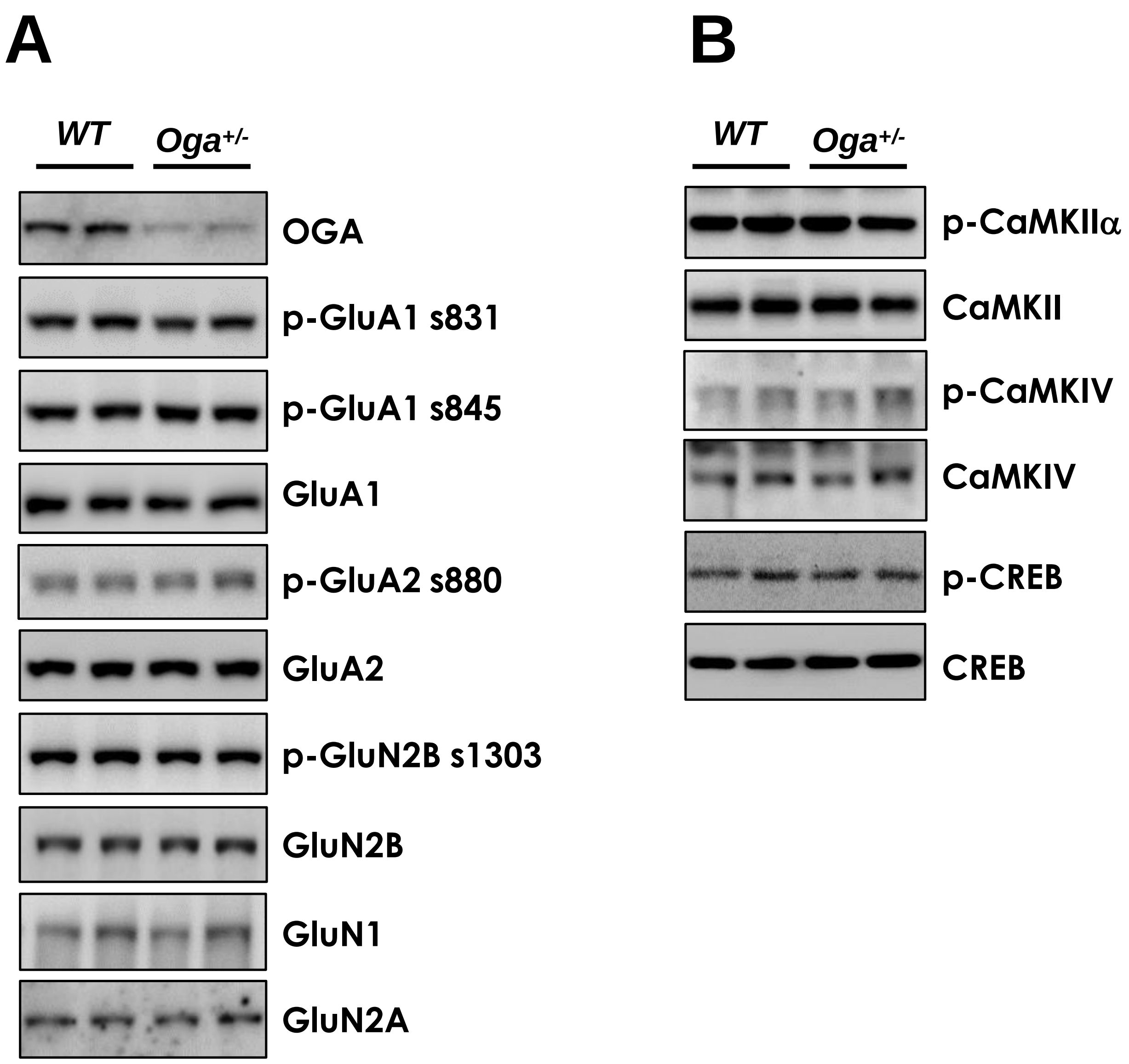

Figure S2. *Oga* heterozygosity does not affect total protein levels and phosphorylation of AMPA and NMDA receptor subunits and phosphorylation of CaMKII, CaMKIV, and CREB in hippocampus (A) Representative immunoblots of phospho and total protein levels of AMPA and NMDA receptor (B) Representative immunoblots of phospho and total protein levels of CaMKII, CaMKIV, and CREB from WT and *Oga*$^{+/-}$ hippocampal lysates.

Supplementary Figure S3

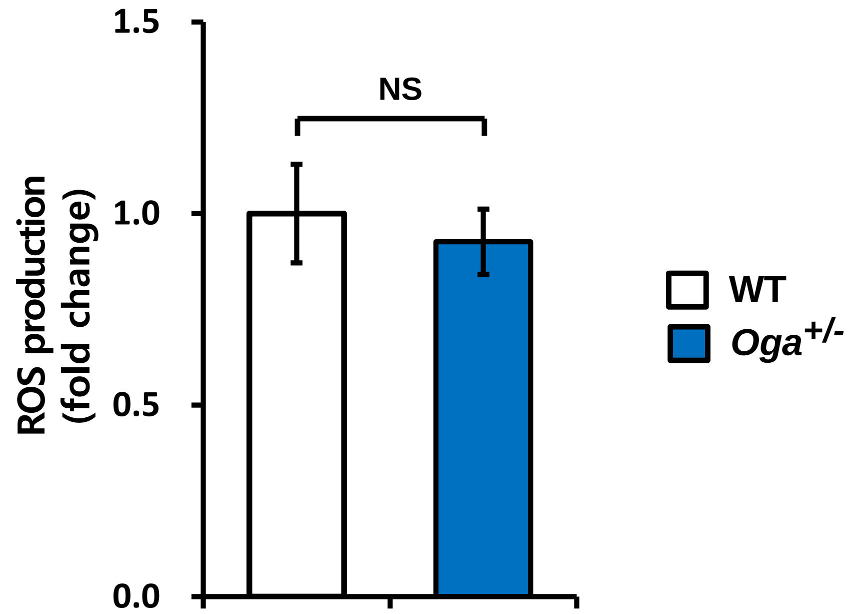

Figure S3. There was no significant difference ROS levels between WT and *Oga*$^{+/-}$ hippocampus. ROS was determined in WT and *Oga*$^{+/-}$ hippocampal homogenates. (WT, n = 6; *Oga*$^{+/-}$, n = 6; unpaired *t*-test, not significant)

Supplementary Figure S4. Full unedited blots.

Figure 1b

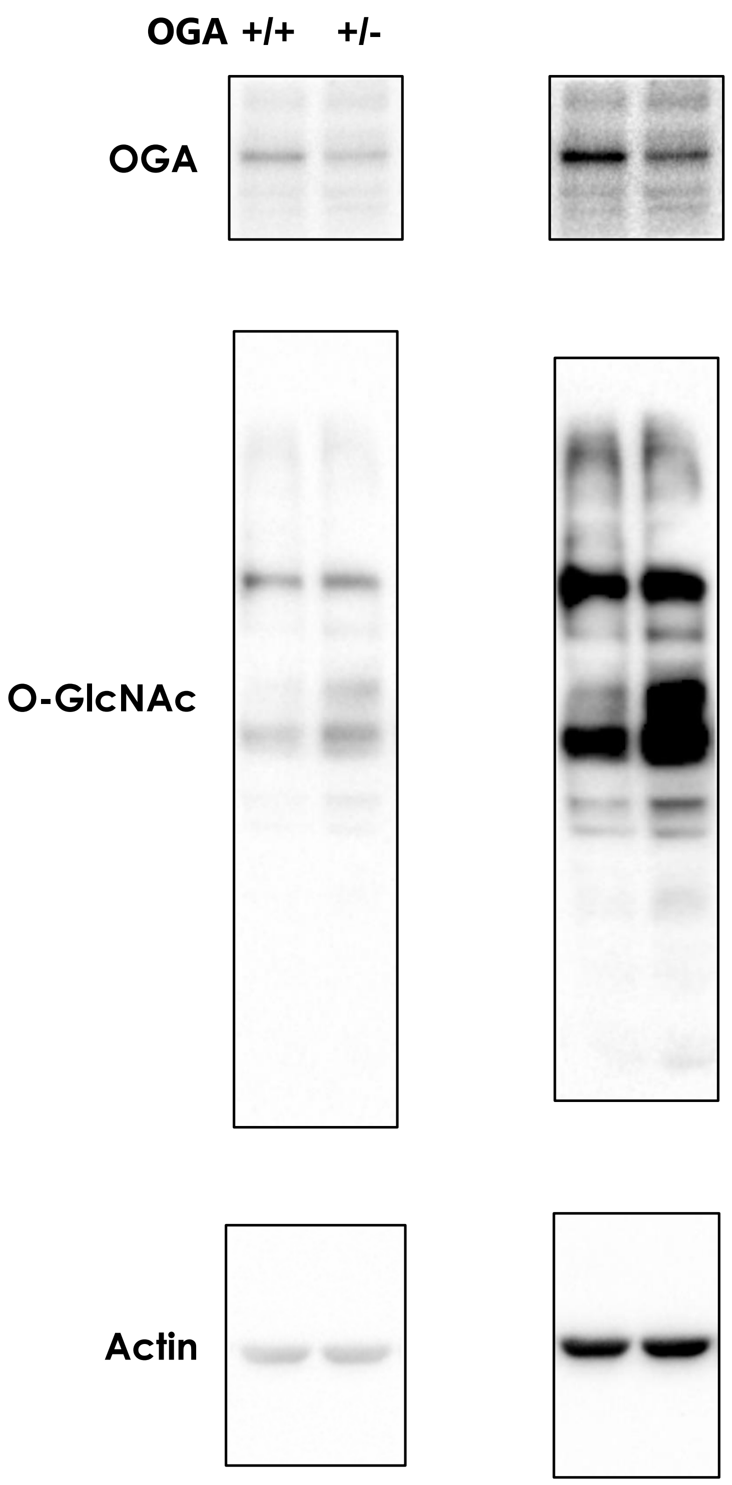

Supplementary Figure S4 continued.

Figure 5a

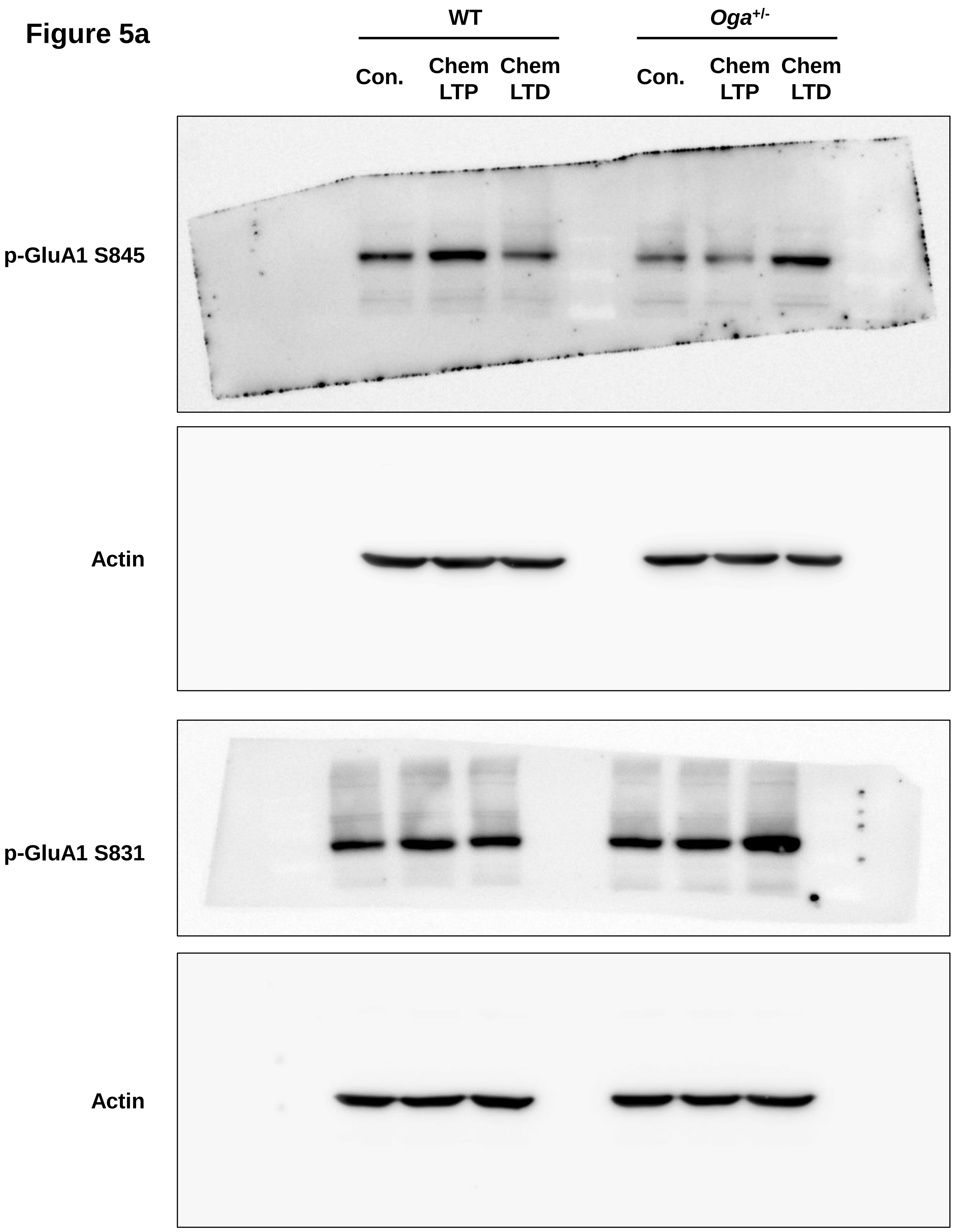

Supplementary Figure S4 continued.

Figure 5a

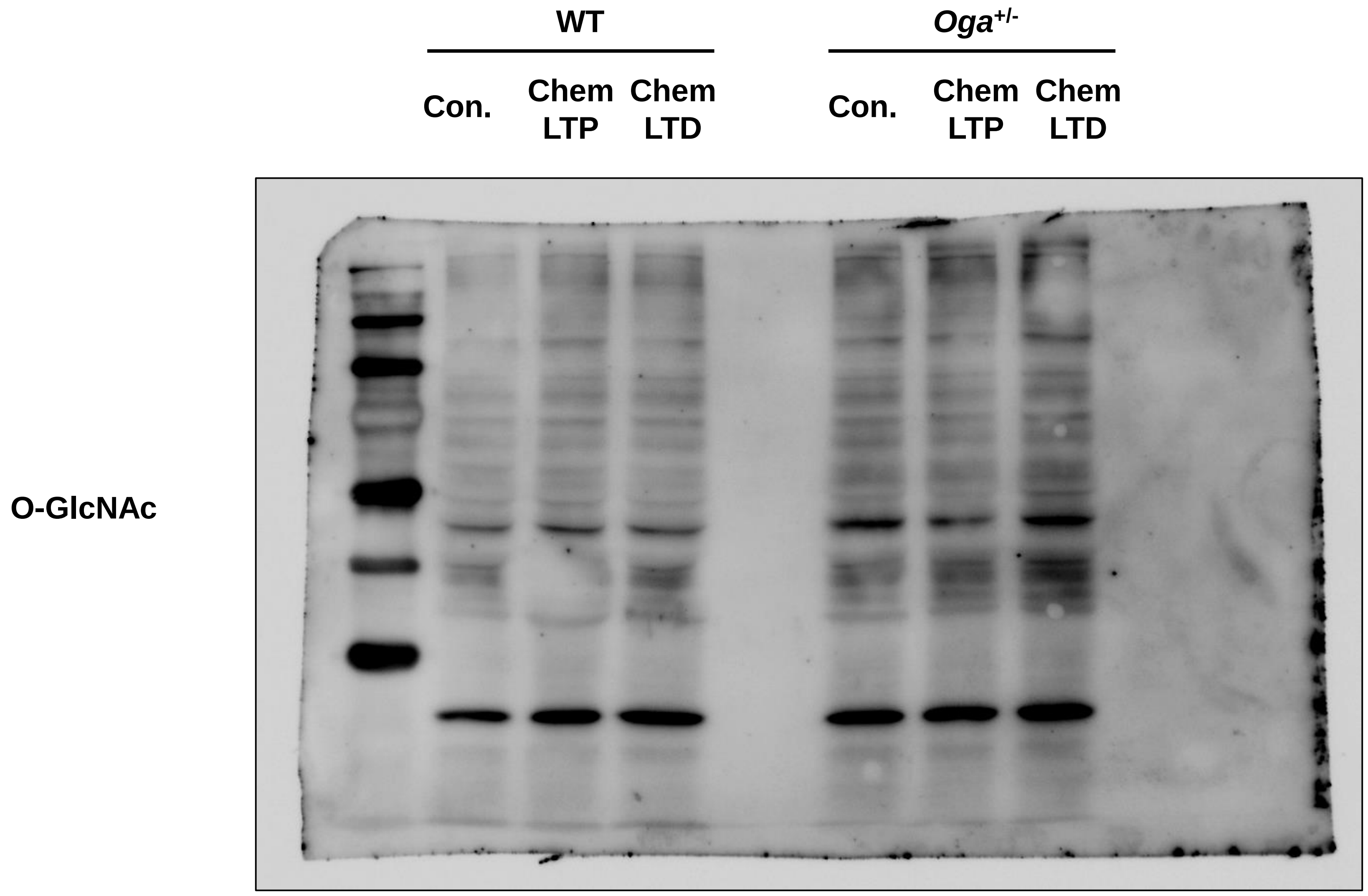

Supplement: Supplemental Information and Figures [file srep44921-s1.pdf]
